# *In-Silico* Determination of Insecticidal Potential of *Vip3Aa-Cry1Ac* Fusion Protein Against *Lepidopteran* Targets Using Molecular Docking

**DOI:** 10.3389/fpls.2015.01081

**Published:** 2015-12-02

**Authors:** Aftab Ahmad, Muhammad R. Javed, Abdul Q. Rao, Muhammad A. U. Khan, Ammara Ahad, Salah ud Din, Ahmad A. Shahid, Tayyab Husnain

**Affiliations:** ^1^Center of Excellence in Molecular Biology, University of the PunjabLahore, Pakistan; ^2^Department of Bioinformatics and Biotechnology, Government College University FaisalabadFaisalabad, Pakistan

**Keywords:** Vip3Aa, Cry1Ac, fusion protein, *Lepidopteran*, homology modeling, molecular docking

## Abstract

*Study and research of Bt (Bacillus thuringiensis*) transgenic plants have opened new ways to combat insect pests. Over the decades, however, insect pests, especially the *Lepidopteran*, have developed tolerance against *Bt* delta-endotoxins. Such issues can be addressed through the development of novel toxins with greater toxicity and affinity against a broad range of insect receptors. In this computational study, functional domains of *Bacillus thuringiensis* crystal delta-endotoxin (Cry1Ac) insecticidal protein and vegetative insecticidal protein (Vip3Aa) have been fused to develop a broad-range Vip3Aa-Cry1Ac fusion protein. Cry1Ac and Vip3Aa are non-homologous insecticidal proteins possessing receptors against different targets within the midgut of insects. The insecticidal proteins were fused to broaden the insecticidal activity. Molecular docking analysis of the fusion protein against aminopeptidase-N (APN) and cadherin receptors of five *Lepidopteran* insects (*Agrotis ipsilon, Helicoverpa armigera, Pectinophora gossypiella, Spodoptera exigua*, and *Spodoptera litura*) revealed that the Ser290, Ser293, Leu337, Thr340, and Arg437 residues of the fusion protein are involved in the interaction with insect receptors. The *Helicoverpa armigera* cadherin receptor, however, showed no interaction, which might be due to either loss or burial of interactive residues inside the fusion protein. These findings revealed that the Vip3Aa-Cry1Ac fusion protein has a strong affinity against *Lepidopteran* insect receptors and hence has a potential to be an efficient broad-range insecticidal protein.

## Introduction

Cotton (*Gossypium hirsutum*) is among the most important crops worldwide, cultivated in more than 80 countries. In addition to the use of cotton in the textile industry, it also has applications in the seed-oil, paper, fertilizer and livestock feed industries (Palle et al., 2013). Despite its importance, there are many threats to cotton production, including weeds, insect pests, drought and cotton leaf curl virus (CLCuV). The most severe are insect pests and CLCuV.

According to a 2013-2014 report by Cotton—Statista, the world's top four cotton-producing countries are China, India, USA and Pakistan, which together produce approximately two-thirds of the total global cotton crop (http://www.statista.com/study/15461/cotton-statista-dossier/). China is the leading importer, with a global share of 20%. However, the US is the leading exporter of cotton, with a global share of 42% (Sabir et al., 2011). With advancing technology, the use of genetically modified plants, especially Bt (*Bacillus thuringiensis*) transgenic plants, has opened new channels to control such control problems that arise from insect-pests (Schnepf et al., 1998). The first transgenic cotton with crystal delta-endotoxin insecticidal protein from the gram-positive bacterium *Bacillus thuringiensis* (Cry1Ac) was commercially available in 1995 (Ellsworth et al., 2000), and since then, many new advances have been achieved.

Bt cotton was initially successful in providing protection against *Lepidoptera;* however, over the years, insects such as cotton bollworms have developed resistance against both delta-endotoxins (Cao et al., 2002; Ferré and Van-Rie, 2002; Shelton et al., 2002; Zhao et al., 2002; Tabashnik et al., 2003; Kain et al., 2004). Therefore, to widen the insecticidal activity of pest control programmes and to combat insect resistance, novel toxins with greater toxicity and affinity against a broad range of insect receptors are required.

Vegetative insecticidal protein (Vip3Aa) from *Bacillus thuringiensis* (Bt) is a candidate novel toxin because of its greater toxicity and unique receptor binding sites (Tabashnik et al., 2008). Cry1Ac is produced during the bacterial reproductive growth phase, while Vip3Aa is secreted during the bacterial vegetative growth phase (Wu et al., 2011). Both are non-homologous insecticidal proteins possessing different receptors within the midgut of *Lepidoptera* with unique insecticidal impact (Yu et al., 1997; Lee et al., 2003).

Vip3Aa protein is toxic to black cutworm (BCW), fall armyworm (FAW), and European corn borer (ECB) (Lemes et al., 2014). Vip3Aa at a concentration of 140 ng/ml in the diet exhibited a 100% mortality rate against FAW, BCW, and beet armyworm while Cry1Ac showed a lower effect against these insects. Cry1Ac, however, is more toxic to *Pectinophora gossypiella* (Saunders), *Heliothis virescens* (Fabricius) and *Helicoverpa zea* (Boddie) (Krishnamoorthy et al., 2007). Vip3Aa protein is more toxic and has a broader affinity than Cry1Ac. The separate use of these insecticidal proteins has limited their spectrum and receptor affinity. Hence, there is still a need to broaden the spectrum and receptors of insecticidal proteins to target as many chewing insects as possible (Sivasupramaniam et al., 2008). Previously, fusion protein was used in cotton plants to increase the concentration of Vip3A protein by producing chimeric Tvip3A^*^ protein. Transgenic plants possessing Tvip3A^*^ genes showed an approximately 100% mortality rate in case of beet armyworm and FAW (Wu et al., 2011).

Hence, in this study, an effort was made to devise a codon-optimized broad-spectrum fusion protein of Cry1Ac and Vip3Aa to combat insect resistance. The fusion protein may provide a combined insecticidal effect against the insects targeted by the separate proteins and thus will make the toxin more powerful. The insect receptor binding of the fusion protein model has been evaluated through docking assays.

## Materials and methods

### Sequence retrieval and modeling of fusion protein

The full-length amino acid sequences of Cry1Ac protein (Accession # ACC86135.1, GI # 186694306) and Vip3Aa protein (Accession # ABX90027.1, GI# 162424669) were retrieved from NCBI and their Ramachandran plot were created by the online tool RAMPAGE (http://mordred.bioc.cam.ac.uk/~rapper/rampage.php; Figure S1 in Supplementary Material). The functional domains of both sequences were determined using the InterPro tool available on the EBI web page (www.ebi.ac.uk/interpro/). The delta endotoxin and galactose binding domains (3-616 amino acids) of Cry1Ac were fused with the vegetative insecticidal protein 3A (12-188 amino acids) and galactose binding (536-654 amino acids) domains of Vip3Aa. To prepare the fusion protein sequence, amino acid residues of all the functional domains of both proteins were combined together and were again evaluated through InterPro to determine their presence, before subjecting to three-dimensional modeling using the online I-TASSER server (http://zhanglab.ccmb.med.umich.edu/I-TASSER/). This Bioinformatics tool produced a model of the fusion protein based on homology modeling and threading. For homology modeling, the PDB templates used were 4W8J (identity 71% and coverage 80%) and 1CIY (identity 75% and coverage 52%) for Cry1Ac and Vip3Aa, respectively.

### Refinement, evaluation, and validation of fusion protein model

The model was further refined using the ModRefiner online tool accessed through the Zhang Lab website (http://zhanglab.ccmb.med.umich.edu/ModRefiner/). This tool minimized the energy of the model and moved the residues of protein within the allowed region. The fusion protein model was evaluated and validated by a Ramachandran plot and by determining the physiochemical properties. The Ramachandran plot was created by the online tool RAMPAGE (http://mordred.bioc.cam.ac.uk/~rapper/rampage.php), which determined the stereochemical properties of the fusion protein to examine its stability. The hydropathy plot and physiochemical properties were determined using the ProtScale online tool available from EXPASY (http://web.expasy.org/protscale/).

### Primary structure analysis of fusion protein model

For the computation of various physical and chemical parameters of the fusion protein model, the ProtParam tool (http://web.expasy.org/protparam/) was used. The tool provided the molecular weight, theoretical isoelectric point (p*I*), estimated half-life and instability index of the fusion protein model.

### Modeling of receptor molecules for docking analysis

Protein sequences of the aminopeptidase-N (APN) and cadherin receptors of five *Lepidopteran* insects: a BCW, *Agrotis ipsilon* (Accession # AAP33525.1 and AEB97396.1, respectively); a cotton bollworm, *Helicoverpa armigera* (Accession # AAN04900.1 and AFB74174.1, respectively); a pink bollworm, *Pectinophora gossypiella* (Accession # AIA80582.1 for cadherin); a beet armyworm, *Spodoptera exigua* (Accession # AAT99437.1 and AGS80251.1, respectively) and a cotton leaf worm, *Spodoptera litura* (Accession # AAK69605.1 and AFJ04291.1, respectively) were retrieved from NCBI (Bergamasco et al., 2013). The protein sequences of the aminopeptidase-N (APN) and cadherin-like receptors of Cry1Ac and Vip3Aa were also retrieved from NCBI, as their protein structures were not available (Gill et al., 1992; Lee et al., 2003). These sequences were further submitted to the LOMETS tool (http://zhanglab.ccmb.med.umich.edu/LOMETS/) and Phyre2 tool (www.sbg.bio.ic.ac.uk/phyre2/) for modeling. These tools were used due to the sequence length submission limitation on the I-TASSER server. A phylogenetic tree of the receptor proteins was also created by using MEGA6 software, freely available desktop application (Figure S2 in Supplementary Material). The predicted models were then refined, validated and evaluated using ModRefiner, RAMPAGE and ProtScale.

### Protein-protein docking analysis of fusion protein and receptor molecules

After validation of the fusion protein and receptor models, protein-protein docking of all the receptor models with the fusion protein was conducted using the Z-DOCK server (http://zdock.umassmed.edu/). The Z-DOCK server is best known for protein-protein docking. This server provides 10 top predicates in the form of PDB files. The interface of Z-DOCK provides information about the bonds between the ligand (insect receptor proteins) and receptor (Cry1Ac-Vip3Aa fusion protein). It also provides files containing information regarding the interactions. To find the interactions between the ligand and receptor proteins, the PDBePISA online tool (http://www.ebi.ac.uk/pdbe/pisa/) was used. The PDB viewer was used for the visualization of structures showing the interactions between the ligand and receptors.

## Results

### Modeling, refinement, evaluation, and validation of fusion protein

**Figure 1:**
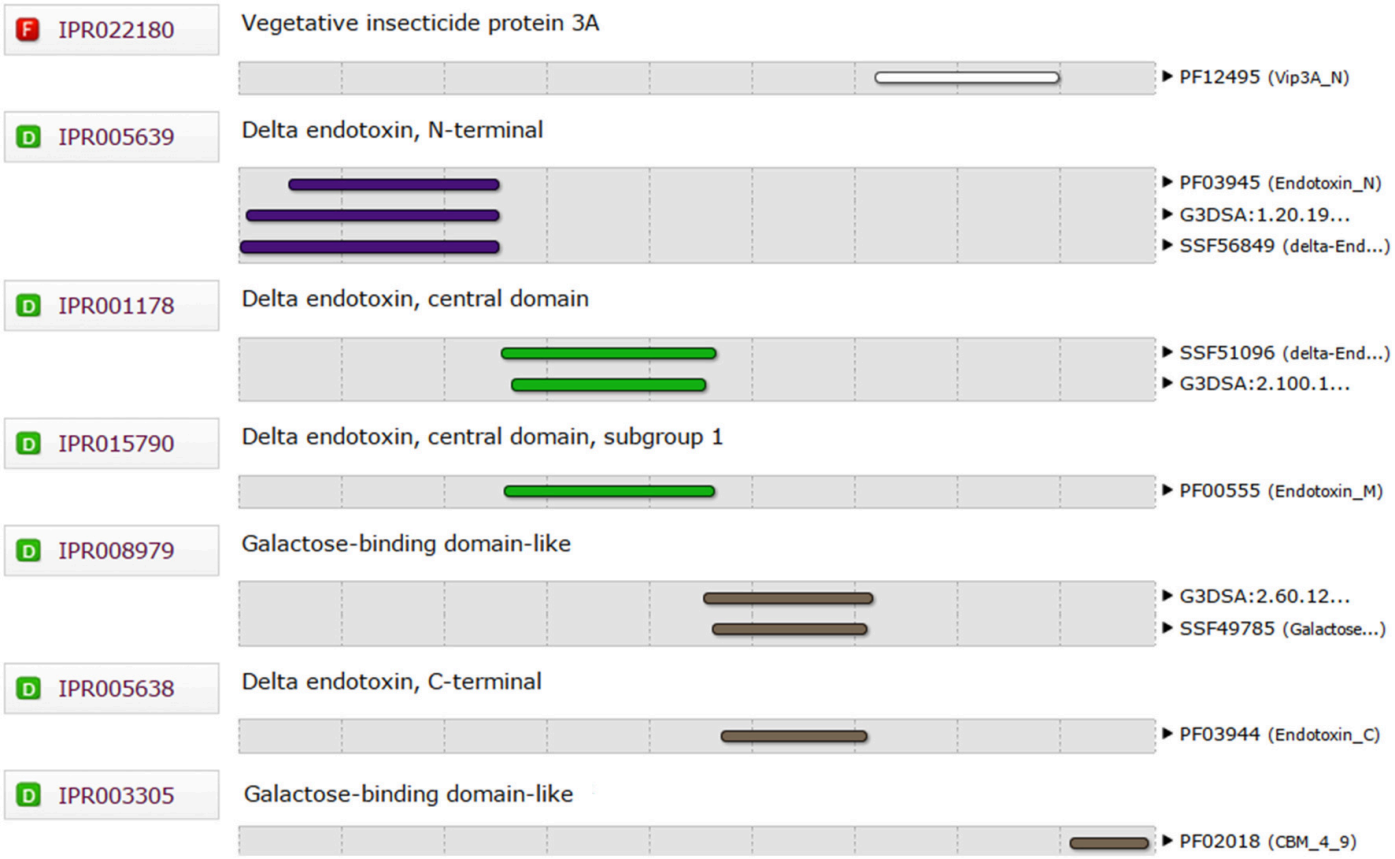
**Functional domains analysis of Cry1Ac-Vip3Aa fusion protein sequence through InterPro**.

**Figure 2:**
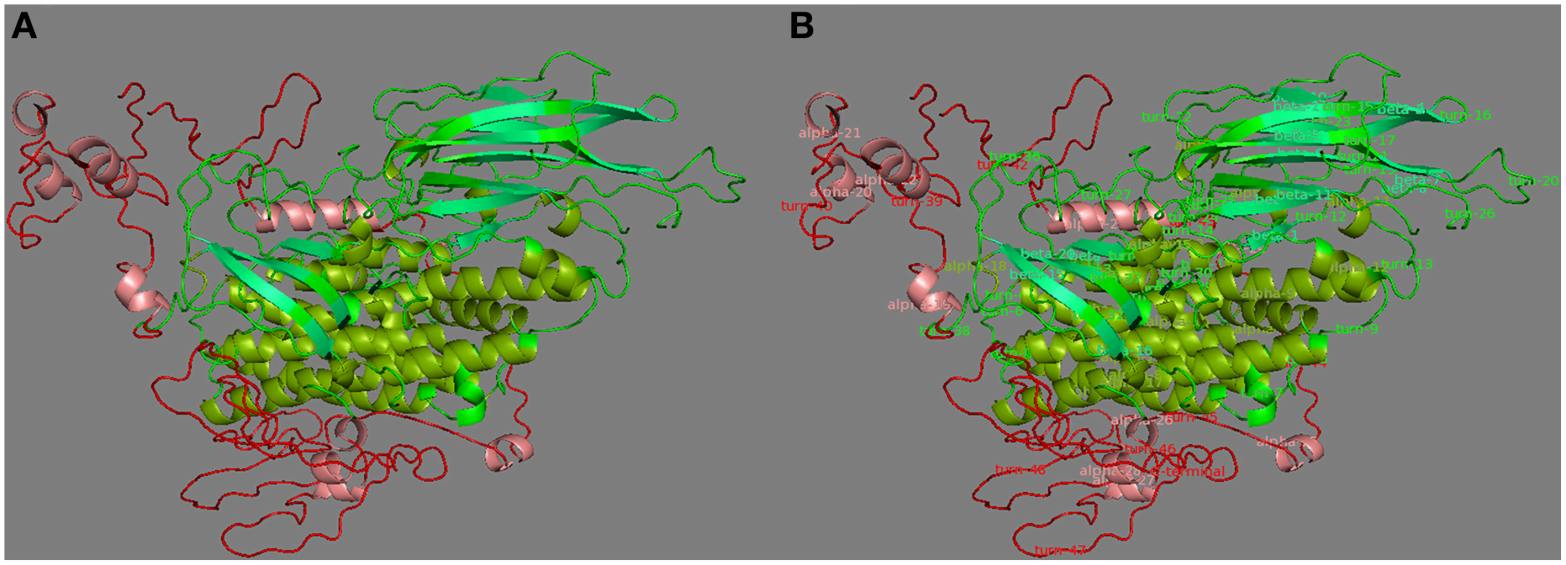
**(A)** Three dimensional protein model of Cry1Ac-Vip3Aa fusion protein, predicted by I-TASSER. Where, green and red represent Cry1Ac and Vip3A domains, respectively. **(B)** Secondary structure labeled 3D Cry1Ac-Vip3Aa fusion protein model. Where, the α-helices and β-sheets of Cry1Ac are represented by split-pea and lime-green colors, respectively. Whereas, the α-helices of Vip3Aa are represented by salmon color.

**Figure 3:**
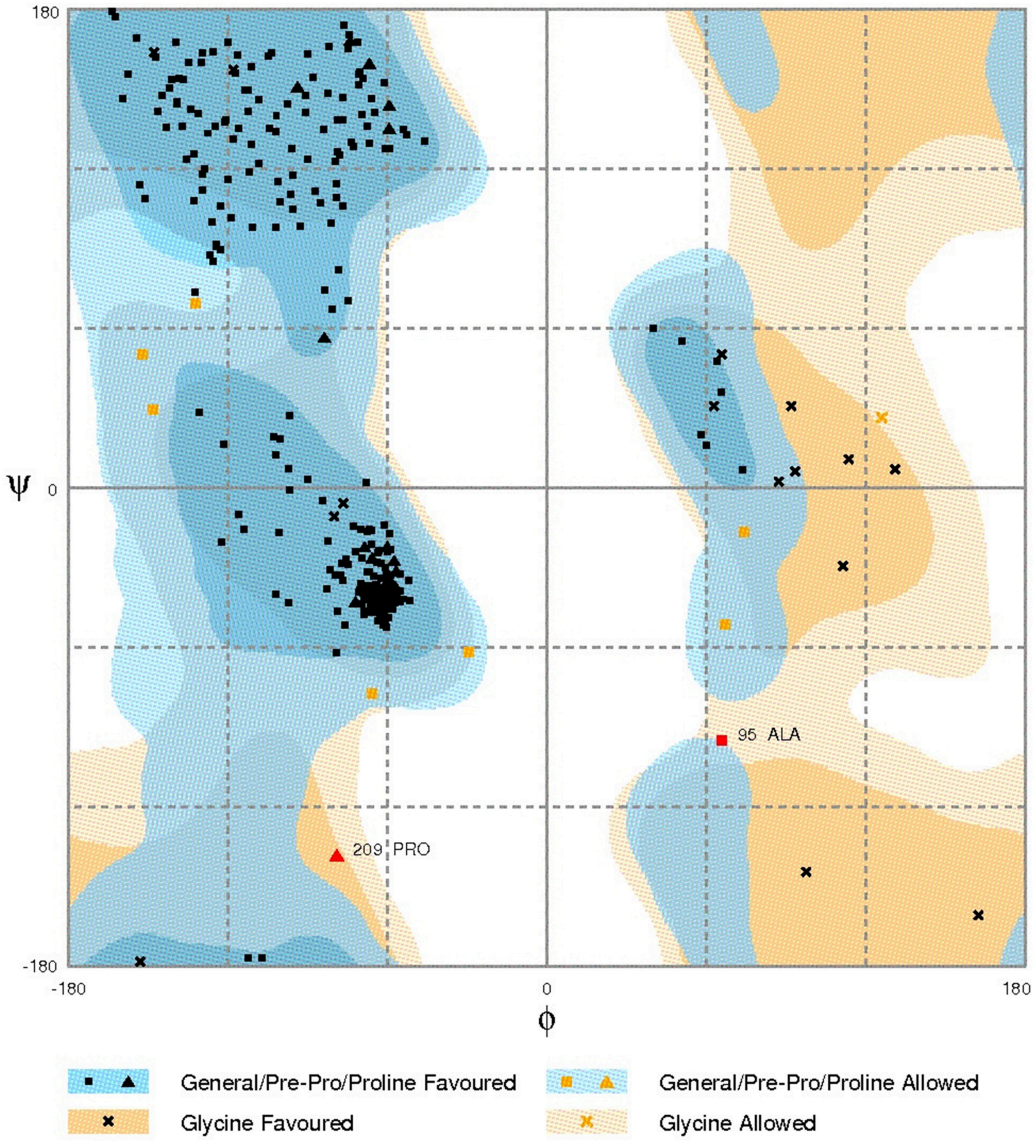
**Ramachandran plot analysis of Cry1Ac-Vip3Aa fusion protein model to visualize dihedral angles; φ against ψ**. At the bottom of the image the summary of evaluating residues is presented.

The functional domains of Cry1Ac-Vip3Aa fusion protein sequence were defined using the InterPro tool (Figure 1), which was further subjected to modeling. The 3D model of the functional domains of the fusion protein was constructed using the online I-TASSER server and then refined by ModRefiner (Figure 2). The resulted Cry1Ac-Vip3Aa fusion protein model consisted of 892 residues (Cry1Ac; 1-616 residues and Vip3Aa; 617-892 residues). The predicted model was evaluated using a Ramachandran plot constructed by RAMPAGE (Figure 3). The Ramachandran plot illustrated that 787 (88.2%) residues of the predicted model were in the favored region, 77 (8.7%) in the allowed region, and 28 (3.1%) in the outlier region. As more than 90% of the residues of the predicted model were in the favored and allowed regions, the model was well validated.

### Primary structure analysis of fusion protein model

Primary structure analysis of the fusion protein model was performed using ProtParam. The fusion protein has a total length of 892 amino acids and theoretical p*I* of 5.49. The instability index was 34.75, classifying it as a stable protein. The estimated half-life in mammalian reticulocytes was 30 h, while in yeast and *Escherichia coli* was more than 20 and 10 h, respectively.

### Protein-protein docking of fusion protein and receptor molecules

#### Docking analysis of *Agrotis ipsilon* aminopeptidase-N (APN) and cadherin receptors with fusion protein

**Figure 4:**
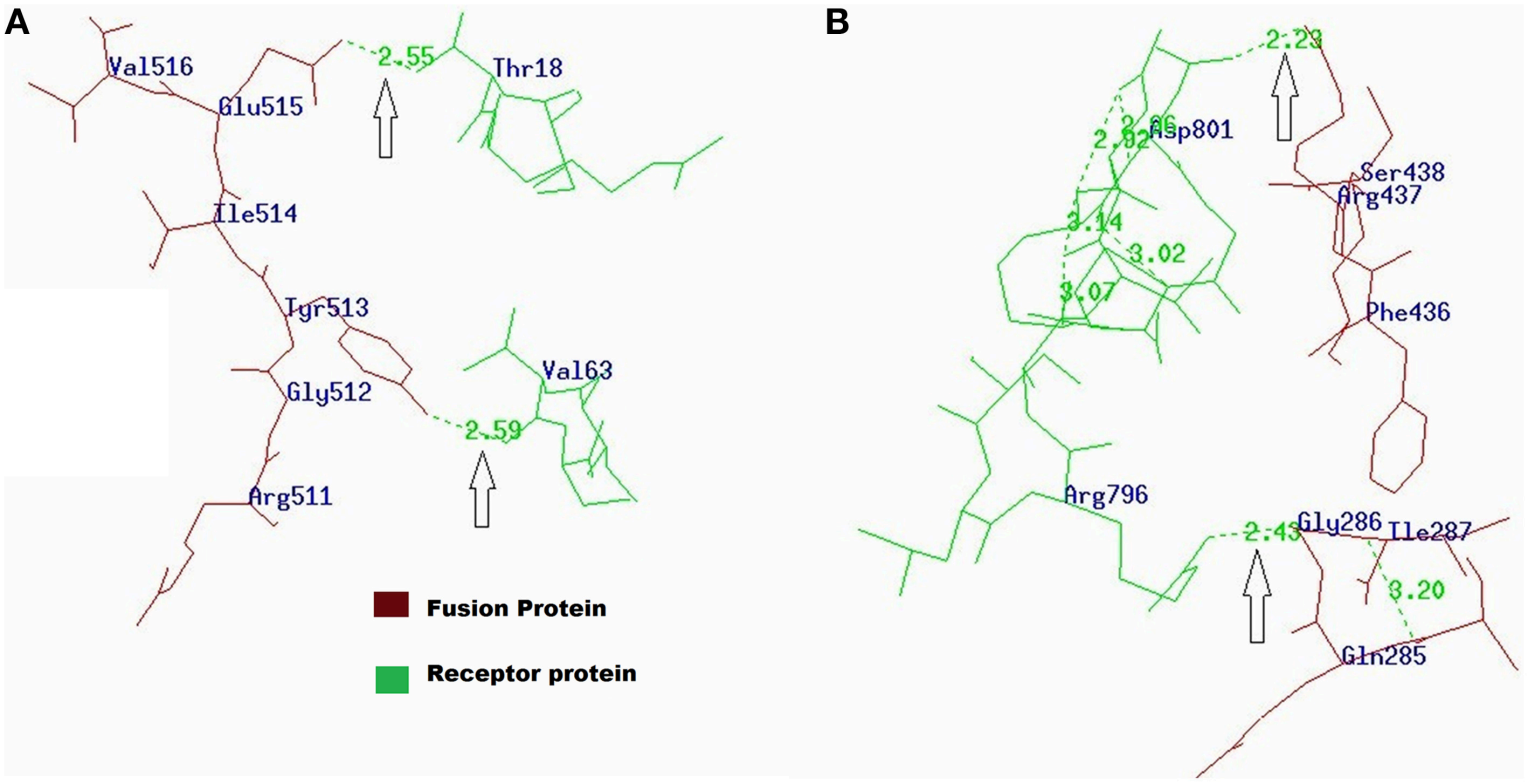
**(A)** Interactions of fusion protein with *Agrotis ipsilon* APN receptor visualized by PDBViewer. Tyr513 and Glu515 residues of fusion protein were interacting with Val63 and Thr18 residues of *Agrotis ipsilon* APN receptor. **(B)** Interaction of fusion protein with *Agrotis ipsilon* cadherin receptor. The Arg437 and Gly286 residues of fusion protein were interacting with Asp801 and Arg796 residues of *Agrotis ipsilon* cadherin receptor.

The amino acids and their molecules involved in interchain H-bonds within the predicted docking models generated by the ZDOCK server were evaluated using PDBePISA. The tool helped to find the best interactions between the fusion protein and *Agrotis ipsilon* aminopeptidase-N (APN) or cadherin receptors. The results showed that the Tyr513 and Glu515 residues of Cry1Ac-Vip3Aa fusion protein were interacting with the Val63 and Thr18 residues of the *Agrotis ipsilon* APN receptor (Table 1; Figure 4A). In the case of fusion protein interactions with the *Agrotis ipsilon* cadherin receptor, the Arg437 and Gly286 residues of the fusion protein were expected to interact with the Asp801 and Arg796 residues of the *Agrotis ipsilon* cadherin receptor (Table 2; Figure 4B). These findings indicated that the Cry1Ac-Vip3Aa fusion protein has a strong affinity with the *Agrotis ipsilon* receptors and thus can be used against the species as an efficient insecticidal protein.

**Table 1:** **Interaction of fusion protein with ***Agrotis ipsilon*** APN receptor**.

**Sr. No**.	**Fusion protein**	**Dist. [Å]**	***Agrotis ipsilon* APN receptor**
1	A: Ser 329 [OG]	3.44	: Thr 18 [OG1]
2	A: Gln 701 [NE2]	3.69	: Glu 41 [OE2]
3	A: Asn 507 [ND2]	3.59	: Phe 61 [O]
4	A: Tyr 513 [OH]	2.59	: Val 63 [O]
5	A: Ser 504 [OG]	3.57	: Pro 134 [O]
6	A: Asn 591 [ND2]	3.88	: Asp 135 [OD2]
7	A: Asn 506 [N]	3.01	: Asp 135 [O]
8	A: Ser 329 [O]	3.70	: Thr 18 [OG1]
9	A: Glu 515 [OE2]	2.55	: Thr 18 [OG1]
10	A: Gly 330 [O]	3.82	: Arg 45 [NH2]
11	A: Ile 508 [O]	3.17	: Val 63 [N]
12	A: Ser 504 [O]	3.12	: Phe 136 [N]

*Out of 12 hydrogen bonds present in the docked complex two were less than 3 Armstrong in the distance (highlighted)*.

**Table 2:** **Interaction of fusion protein with ***Agrotis ipsilon*** cadherin receptor**.

**Sr. No**.	**Fusion protein**	**Dist. [Å]**	***Agrotis ipsilon* cadherin receptor**
1	A:Ser 290[OG]	3.74	:Gly 802[O]
2	A:Gln 320[NE2]	3.78	:Glu 675[OE1]
3	A:Phe 335[N]	3.72	:Tyr 588[OH]
4	A:Thr 340[OG1]	3.77	:Asp 673[OD1]
5	A:Thr 340[OG1]	2.84	:Asp 801[OD1]
6	A:Thr 340[OG1]	3.00	:Asp 801[OD2]
7	A:Arg 437[NH1]	2.23	:Asp 801[OD2]
8	A:Gly 286[O]	2.43	:Arg 796[NH2]
9	A:Arg 289[O]	3.64	:Asn 625[ND2]
10	A:Ser 290[OG]	3.80	:Leu 804[N]
11	A:Asp 308[OD1]	3.67	:Arg 740[NH1]
12	A:Ala 309[O]	3.83	:Arg 742[NH2]
13	A:Phe 335[O]	2.85	:Tyr 588[OH]
14	A:Leu 337[O]	2.69	:Trp 674[NE1]

*Out of 14 hydrogen bonds present in the docked complex two were less than 2.5 Å in the distance (highlighted)*.

#### Docking analysis of *Helicoverpa armigera* aminopeptidase-N (APN) and cadherin receptors with fusion protein

The determination of the interactions between fusion protein and *Helicoverpa armigera* aminopeptidase-N (APN) receptor revealed the binding of the Arg526 and Ser293 residues of the Cry1Ac-Vip3Aa fusion protein with the Thr902 and Lys47 residues of *Helicoverpa armigera* APN receptor (Table S1 and Figure S3A in Supplementary Material). In the model generated for the docking of the fusion protein and *Helicoverpa armigera* cadherin receptor, however, no interacting residues were observed. These findings indicated that due to the fusion of the Cry1Ac and Vip3Aa protein molecules, the amino acid residues that would interact with the *Helicoverpa armigera* cadherin receptor were either lost or buried inside the fusion protein model and thus were not available for interaction.

#### Docking analysis of *Pectinophora gossypiella* cadherin receptor with fusion protein

The interactions generated through the ZDOCK server between the fusion protein and *Pectinophora gossypiella* cadherin receptor, as described for the *Agrotis ipsilon and Helicoverpa armigera* receptors, showed the binding of the Asn343 and Ile350 residues of the Cry1Ac-Vip3Aa fusion protein with the Asp478 and Pro604 residues of the *Pectinophora gossypiella* cadherin receptor (Table S2 and Figure S3B in Supplementary Material).

#### Docking analysis of *Spodoptera exigua* aminopeptidase-N (APN) and cadherin receptors with fusion protein

The amino acids and molecules involved in interchain H-bonds, without selecting any residue in the fusion protein, were evaluated using the ZDOCK server as described earlier. The PDBePISA tool was used to find the best interactions between fusion protein and the *Spodoptera exigua* aminopeptidase-N (APN) and cadherin receptors. The results showed that a number of residues of the Cry1Ac-Vip3Aa fusion protein are expected to interact with the *Spodoptera exigua* receptors. Among these interacting residues, Asn507 and Ser290 of the fusion protein showed interaction with the Tyr513 and Asp576 residues of the *Spodoptera exigua* APN receptor (Table S3 and Figure S3C in Supplementary Material). In the case of the interactions of the fusion protein with the *Spodoptera exigua* cadherin receptor, the Leu337 and Thr340 residues were expected to interact with the Asp674 and Asp799 residues of the *Spodoptera exigua* cadherin receptor (Table S4 and Figure S4A in Supplementary Material). These findings further outlined the strong affinities of *Spodoptera exigua* receptors with the Cry1Ac-Vip3Aa fusion protein, which can therefore also be used as an efficient insecticidal protein against this species.

#### Docking analysis of *Spodoptera litura* aminopeptidase-N (APN) and cadherin receptors with fusion protein

The docking analysis results with *Spodoptera litura* receptors also showed that the Ser290 and Ser293 residues of the fusion protein are expected to interact with the Phe877 and Thr885 residues of the *Spodoptera litura* APN receptor (Table S5 and Figure S4B in Supplementary Material). In the case of the *Spodoptera litura* cadherin receptor, the Tyr313 and Tyr306 residues of fusion protein were expected to interact with the Tyr490 and Gln626 residues of the *Spodoptera litura* cadherin receptor (Table S6 and Figure S4C in Supplementary Material). These findings revealed that the fusion protein can also act as an efficient insecticidal protein against *Spodoptera litura*.

## Discussion

The Bt cotton planting area has progressively increased since 1995, particularly in China and India. Bt cotton was initially successful in providing protection against *Lepidoptera;* however, over the years, insects such as cotton bollworms have developed resistance against both delta-endotoxins (Cao et al., 2002).

In this study, a Cry1Ac-Vip3Aa fusion protein and its docking against five *Lepidopteran* species' (*Agrotis ipsilon, Helicoverpa armigera, Pectinophora gossypiella, Spodoptera exigua*, and *Spodoptera litura*) aminopeptidase-N (APN) and cadherin receptors have been modeled. The Cry1Ac protein is 1178 amino acids long. Its protoxin is 135 kDa, while the activated protein is only 65 kDa (approximately). The Vip3Aa protein is 789 amino acids long with a molecular mass of 88 kDa (Schnepf et al., 1998).

In the current study, the Ser504, Asn506, Asn507, and Ile508 residues of the Cry1Ac protein within the Cry1Ac-Vip3Aa fusion protein showed interactions with insect receptors. These results are consistent with the reported interacting residues of Cry1Ac protein. Earlier protein-protein docking results also reported that the interacting residues of Cry1Ac domain III are within the range 503–525 (Avisar et al., 2004). It was also known that domain III of the Cry1Ac protein has some residues, namely 503–513, including S503, S504, 506NNI508, 509QNR511, 522ST523, and 524ST525, which are responsible for interaction with *Lepidopteran* insects (Lee et al., 1999). The residues including Q509, N510, R511, Y513, and W545 form a binding site that can interact with insect proteins, as reported previously (Sengupta et al., 2013). Arg368 and 369 of Cry1Ac were also reported to be involved in the interaction of the toxin protein with insect midgut protein (Lee et al., 2000). Additionally, in this study, some novel residues such as Ser290, Ser293, Arg289, Glu332, Leu337, Gly339, Thr340, and Arg437 were found to possess interacting properties in most of the docked results.

Within the Cry1Ac-Vip3Aa fusion protein, the Lys860, Thr861, Leu863, Gly864, and Gln701 residues of Vip3Aa were also found to be involved in interaction with insect receptors, as summarized in the tables. These results were consistent with the findings of Chen et al. (2010) who successfully transformed the fused Cry1Ab/Vip3H protein into six varieties of transgenic rice to target the Asiatic rice borer, Chilo, and the stem borer *Sesamia inferens*. Yang has shown 100% mortality rate in the targeted insects. Furthermore, Chang et al. (2013) also transformed the Cry1Ab/Cry2Aj and CrylAb/Vip3DA fusion proteins into maize to target Asian corn borer-BtR and Asian corn borer-BtS. Chang et al. (2013) achieved 95% mortality of the targeted insects in the reported attempt.

Hence, the Cry1Ac-Vip3Aa fusion protein has strong potential as a broad-spectrum insecticidal protein. The findings of this study can also be validated by transforming the fusion protein into the cotton plant shoot apex by *Agrobacterium*, as reported earlier (Rao et al., 2009). For this purpose, the fusion protein can be reverse translated, and a gene cassette can be designed for transformation into cotton embryos through the *Agrobacterium* transformation method.

## Author contributions

Aftab Ahmad performed the experiments and contributed in manuscript writing and revision. Muhammad R. Javed conceived and designed the experiments. Abdul Q. Rao and Tayyab Husnain technically contributed in manuscript writing. Ammara Ahad and Muhammad A. U. Khan helped in data analysis. Sala hud Din and Ahmad A. Shahid carried out manuscript revision.

### Conflict of interest statement

The authors declare that the research was conducted in the absence of any commercial or financial relationships that could be construed as a potential conflict of interest.

## Supplementary material

Figure S1 **(A)** Ramachandran plot analysis of Cry1Ac protein model to visualize dihedral angles; φ against ψ. **(B)** Ramachandran plot analysis of Vip3Aa protein model to visualize dihedral angles; φ against ψ.

Figure S2 **Phylogenetic tree based on protein sequences of the aminopeptidase-N (APN) and cadherin receptors of five *Lepidopteran* insects**. The evolutionary history was inferred using the Neighbor-Joining method. The optimal tree with the sum of branch length = 4.07705262 is shown. Bootstrap values expressed as a percentage of 1000 replications, are given at the branching points. The tree is drawn to scale, with branch lengths in the same units as those of the evolutionary distances used to infer the phylogenetic tree. The evolutionary distances were computed using the Poisson correction method and are in the units of the number of amino acid substitutions per site. The scale bar corresponds to 0.2-estimated amino acid substitution per sequence position. The analysis involved 9 amino acid sequences. All positions with less than 95% site coverage were eliminated. That is, fewer than 5% alignment gaps, missing data and ambiguous bases were allowed at any position. There were a total of 132 positions in the final dataset. Evolutionary analyses were conducted in MEGA6 software package. The taxa are representing the amino acid sequences of aminopeptidase-N (APN) and cadherin receptors of five *Lepidopteran* insects along with the accession number (NCBI) of retrieved sequences in parentheses.

Figure S3 **(A)** Interaction of Arg526 and Ser293 residues of fusion protein with Thr902 and Lys47 residues of *Helicoverpa armigera* APN receptor. **(B)** Interaction of Asn343 and Ile350 residues of fusion protein with Asp478 and Pro604 residues of *Pectinophora gossypiella* cadherin receptor. **(C)** Interaction of Asn484 and Ser293 residues of fusion protein with Ser515 and Thr582 residues of the *Spodoptera exigua* APN receptor.

Figure S4 **(A)** Interaction of fusion protein with *Spodoptera exigua* cadherin receptor. The Leu337 and Thr340 residues of fusion protein were interacting with Asp674 and Asp799 residues of the *Spodoptera exigua* cadherin receptor. **(B)** Interaction of Ser290 and Ser293 residues of fusion protein with Phe877 and Thr885 residues of *Spodoptera litura* APN receptor. **(C)** Interaction of fusion protein with *Spodoptera litura* cadherin receptor. The Tyr313 and Tyr306 residues of fusion protein were interacting with Tyr490 and Gln626 residues of *Spodoptera litura* cadherin receptor.

Table S1 **Interaction of fusion protein with *Helicoverpa armigera* aminopeptidase-N (APN) receptor**. Out of 24 hydrogen bonds present in the docked complex two were less than 2.5 Å in the distance (highlighted).

Table S2 **Interaction of fusion protein with *Pectinophora gossypiella* cadherin receptor**. Out of 23 hydrogen bonds present in the docked complex two were less than 2.5 Armstrong in distance (highlighted).

Table S3 **Interaction of fusion protein with *Spodoptera exigua* APN receptor**. Out of 8 hydrogen bonds present in the docked complex two were less than 2.5 Armstrong in the distance (highlighted).

Table S4 **Interaction of fusion protein with *Spodoptera exigua* cadherin receptor**. Out of 8 hydrogen bonds present in the docked complex two were less than 3.5 Armstrong in distance (highlighted).

Table S5 **Interaction of fusion protein with *Spodoptera litura* APN receptor**. Out of 19 hydrogen bonds present in the docked complex two were less than 2.5 Armstrong in the distance (highlighted).

Table S6 **Interaction of fusion protein with *Spodoptera litura* cadherin receptor**. Out of 10 hydrogen bonds present in the docked complex two were less than 3 Armstrong in the distance (highlighted).
