# Supplementary figures and images for: In-Silico Determination of Insecticidal Potential of Vip3Aa-Cry1Ac Fusion Protein Against Lepidopteran Targets Using Molecular Docking
Source: Front Plant Sci. 2015 Dec 2;6:1081. doi: 10.3389/fpls.2015.01081 (PMC4667078; doi:10.3389/fpls.2015.01081)

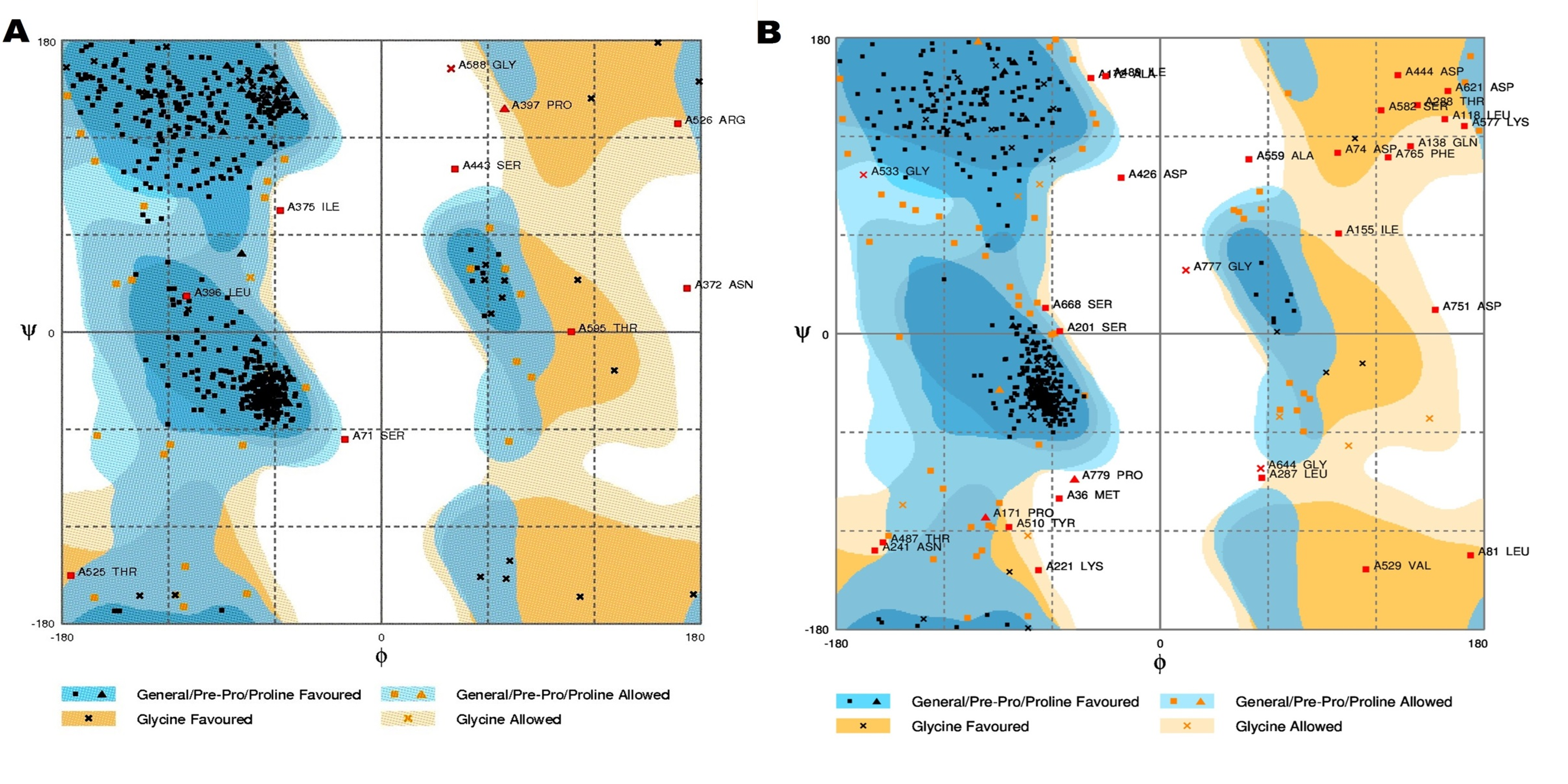

Supplement: Figure S1 — (A) Ramachandran plot analysis of Cry1Ac protein model to visualize dihedral angles; φ against ψ. (B) Ramachandran plot analysis of Vip3Aa protein model to visualize dihedral angles; φ against ψ. [file Image1.TIF]

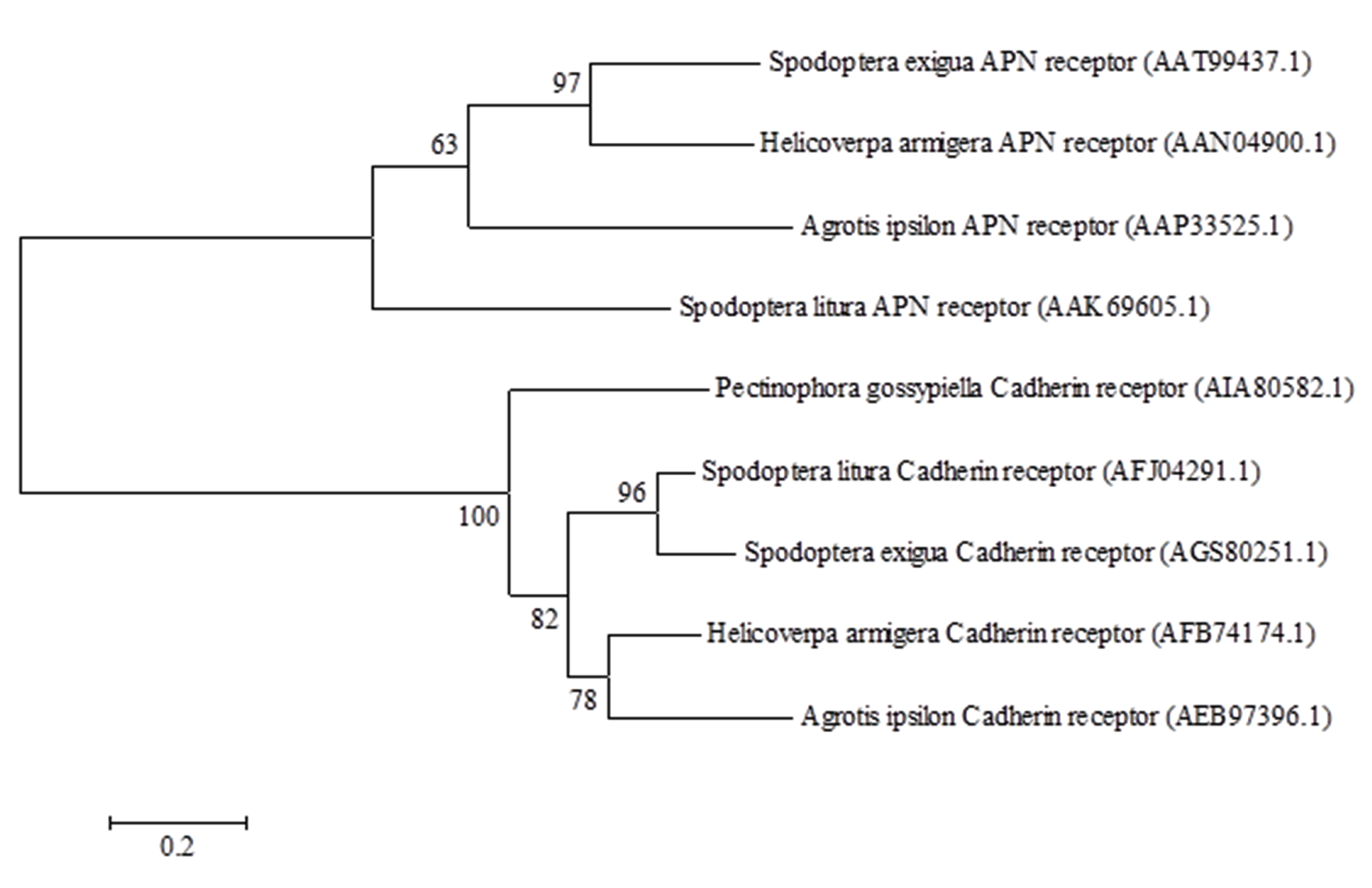

Supplement: Figure S2 — Phylogenetic tree based on protein sequences of the aminopeptidase-N (APN) and cadherin receptors of five Lepidopteran insects. The evolutionary history was inferred using the Neighbor-Joining method. The optimal tree with the sum of branch length = 4.07705262 is shown. Bootstrap values expressed as a percentage of 1000 replications, are given at the branching points. The tree is drawn to scale, with branch lengths in the same units as those of the evolutionary distances used to infer the phylogenetic tree. The evolutionary distances were computed using the Poisson correction method and are in the units of the number of amino acid substitutions per site. The scale bar corresponds to 0.2-estimated amino acid substitution per sequence position. The analysis involved 9 amino acid sequences. All positions with less than 95% site coverage were eliminated. That is, fewer than 5% alignment gaps, missing data and ambiguous bases were allowed at any position. There were a total of 132 positions in the final dataset. Evolutionary analyses were conducted in MEGA6 software package. The taxa are representing the amino acid sequences of aminopeptidase-N (APN) and cadherin receptors of five Lepidopteran insects along with the accession number (NCBI) of retrieved sequences in parentheses. [file Image2.TIF]
