# Supplementary material for: In-Silico Determination of Insecticidal Potential of Vip3Aa-Cry1Ac Fusion Protein Against Lepidopteran Targets Using Molecular Docking
Source: Front Plant Sci. 2015 Dec 2;6:1081. doi: 10.3389/fpls.2015.01081 (PMC4667078; doi:10.3389/fpls.2015.01081)
Supplement: Table S1 — Interaction of fusion protein with Helicoverpa armigera aminopeptidase-N (APN) receptor. Out of 24 hydrogen bonds present in the docked complex two were less than 2.5 Å in the distance (highlighted). [file Table1.DOCX]

**Table-1:** Interaction of fusion protein with *Helicoverpa armigera* aminopeptidase-N (APN) receptor. Out of 24 hydrogen bonds present in the docked complex two were less than 2.5 Å in the distance (highlighted).

| **Sr. No.** | **Fusion protein** | **Dist. [Å]** | | ***Helicoverpa armigera* APN receptor** | |
| --- | --- | --- | --- | --- | --- |
| 1 | A:Thr 340[ OG1] | | 3.25 | | :Gly  37[ O  ] |
| 2 | A:Thr 340[ N  ] | | 3.47 | | :Ala  39[ O  ] |
| 3 | A:Thr 537[ OG1] | | 3.11 | | :Ser  57[ O  ] |
| 4 | A:Arg 526[ NH2] | | 2.48 | | :Thr 902[ OG1] |
| 5 | A:Ser 552[ N  ] | | 3.41 | | :Asn 909[ O  ] |
| 6 | A:Ser 549[ OG ] | | 3.03 | | :Leu 910[ O  ] |
| 7 | A:Phe 551[ N  ] | | 3.80 | | :Leu 910[ O  ] |
| 8 | A:Asn 577[ ND2] | | 2.77 | | :Thr 919[ OG1] |
| 9 | A:Thr 554[ OG1] | | 3.16 | | :Ser 920[ OG ] |
| 10 | A:Gly 339[ O  ] | | 2.62 | | :Ala  39[ N  ] |
| 11 | A:Thr 340[ OG1] | | 2.96 | | :Ala  39[ N  ] |
| 12 | A:Gly 339[ O  ] | | 3.28 | | :Phe  40[ N  ] |
| 13 | A:Ser 293[ OG ] | | 2.30 | | :Lys  47[ N  ] |
| 14 | A:Ser 561[ OG ] | | 3.74 | | :Asn  53[ ND2] |
| 15 | A:Thr 595[ O  ] | | 3.57 | | :Tyr 138[ OH ] |
| 16 | A:Asn 617[ O  ] | | 3.39 | | :Gly 565[ N  ] |
| 17 | A:Glu 615[ OE1] | | 3.14 | | :Ser 567[ N  ] |
| 18 | A:Gly 864[ O  ] | | 2.62 | | :Ser 567[ OG ] |
| 19 | A:Glu 615[ OE2] | | 3.01 | | :Ser 567[ OG ] |
| 20 | A:Leu 863[ O  ] | | 3.07 | | :Arg 569[ N  ] |
| 21 | A:Leu 863[ O  ] | | 3.14 | | :Asn 570[ N  ] |
| 22 | A:Thr 861[ O  ] | | 3.90 | | :Arg 571[ NH1] |
| 23 | A:Lys 862[ O  ] | | 3.61 | | :Arg 571[ NH1] |
| 24 | A:Asn 542[ OD1] | | 3.11 | | :Thr 913[ N  ] |
