# Supplementary material for: In-Silico Determination of Insecticidal Potential of Vip3Aa-Cry1Ac Fusion Protein Against Lepidopteran Targets Using Molecular Docking
Source: Front Plant Sci. 2015 Dec 2;6:1081. doi: 10.3389/fpls.2015.01081 (PMC4667078; doi:10.3389/fpls.2015.01081)
Supplement: Table S2 — Interaction of fusion protein with Pectinophora gossypiella cadherin receptor. Out of 23 hydrogen bonds present in the docked complex two were less than 2.5 Armstrong in distance (highlighted). [file Table2.DOCX]

**Table-2:** Interaction of fusion protein with *Pectinophora gossypiella* cadherin receptor. Out of 23 hydrogen bonds present in the docked complex two were less than 2.5 Armstrong in distance (highlighted).

| **Sr. No.** | **Fusion protein** | **Dist. [Å]** | | ***Pectinophora gossypiella* cadherin receptor** | |
| --- | --- | --- | --- | --- | --- |
| 1 | A:Arg 289[ NH1] | | 3.43 | | :Asn 421[ OD1] |
| 2 | A:Phe 440[ N  ] | | 3.76 | | :Ala 423[ O  ] |
| 3 | A:Phe 440[ N  ] | | 3.49 | | :Glu 424[ O  ] |
| 4 | A:Pro 336[ N  ] | | 3.79 | | :Tyr 429[ OH ] |
| 5 | A:Ser 290[ N  ] | | 2.73 | | :Asp 430[ OD2] |
| 6 | A:Ile 291[ N  ] | | 3.21 | | :Asp 430[ OD2] |
| 7 | A:Tyr 338[ N  ] | | 3.76 | | :Gly 470[ O  ] |
| 8 | A:Gln 320[ NE2] | | 3.29 | | :Asp 473[ OD1] |
| 9 | A:Asn 343[ ND2] | | 2.32 | | :Asp 478[ O  ] |
| 10 | A:Arg 311[ N  ] | | 3.41 | | :Gln 485[ O  ] |
| 11 | A:Arg 437[ NH1] | | 2.61 | | :Ala 505[ O  ] |
| 12 | A:Arg 449[ NH2] | | 2.08 | | :Ala 505[ O  ] |
| 13 | A:Met 341[ N  ] | | 3.74 | | :Glu 506[ OE1] |
| 14 | A:Ile 350[ N  ] | | 2.25 | | :Pro 604[ O  ] |
| 15 | A:Arg 402[ NH1] | | 3.51 | | :Glu 605[ OE2] |
| 16 | A:Ser 290[ OG ] | | 3.59 | | :Phe 428[ N  ] |
| 17 | A:Pro 336[ O  ] | | 2.87 | | :Tyr 429[ OH ] |
| 18 | A:Leu 337[ O  ] | | 3.78 | | :Thr 471[ N  ] |
| 19 | A:Asn 343[ OD1] | | 3.85 | | :Leu 477[ N  ] |
| 20 | A:Gln 378[ OE1] | | 3.87 | | :Asn 486[ ND2] |
| 21 | A:Ala 344[ O  ] | | 3.49 | | :Asn 508[ ND2] |
| 22 | A:Gln 347[ O  ] | | 2.82 | | :Ser 606[ N  ] |
| 23 | A:Gln 348[ O  ] | | 3.64 | | :Ser 606[ N  ] |
