# Supplementary material for: In-Silico Determination of Insecticidal Potential of Vip3Aa-Cry1Ac Fusion Protein Against Lepidopteran Targets Using Molecular Docking
Source: Front Plant Sci. 2015 Dec 2;6:1081. doi: 10.3389/fpls.2015.01081 (PMC4667078; doi:10.3389/fpls.2015.01081)
Supplement: Table S3 — Interaction of fusion protein with Spodoptera exigua APN receptor. Out of 8 hydrogen bonds present in the docked complex two were less than 2.5 Armstrong in the distance (highlighted). [file Table3.DOCX]

**Table-3:** Interaction of fusion protein with *Spodoptera exigua* APN receptor. Out of 8 hydrogen bonds present in the docked complex two were less than 2.5 Armstrong in the distance (highlighted).

| **Sr. No.** | **Fusion protein** | **Dist. [Å]** | | ***Spodoptera exigua* APN receptor** | |
| --- | --- | --- | --- | --- | --- |
| 1 | A:Leu 482[ N ] | | 3.43 | | :Ala 509[ O ] |
| 2 | A:Leu 482[ N ] | | 2.91 | | :Phe 510[ O ] |
| 3 | A:Met 341[ N ] | | 3.51 | | :Tyr 575[ OH ] |
| 4 | A:Ser 290[ OG ] | | 2.64 | | :Asp 576[ OD2] |
| 5 | A:Asn 442[ N ] | | 3.87 | | :Asp 602[ OD2] |
| 6 | A:Gln 320[ OE1] | | 2.94 | | :Lys 610[ NZ ] |
| 7 | A:Asn 507[ OD1] | | 2.93 | | :Tyr 513[ N ] |
