# Supplementary material for: In-Silico Determination of Insecticidal Potential of Vip3Aa-Cry1Ac Fusion Protein Against Lepidopteran Targets Using Molecular Docking
Source: Front Plant Sci. 2015 Dec 2;6:1081. doi: 10.3389/fpls.2015.01081 (PMC4667078; doi:10.3389/fpls.2015.01081)
Supplement: Table S4 — Interaction of fusion protein with Spodoptera exigua cadherin receptor. Out of 8 hydrogen bonds present in the docked complex two were less than 3.5 Armstrong in distance (highlighted). [file Table4.DOCX]

**Table-4:** Interaction of fusion protein with *Spodoptera exigua* cadherin receptor. Out of 8 hydrogen bonds present in the docked complex two were less than 3.5 Armstrong in distance (highlighted).

| **Sr. No.** | **Fusion protein** | **Dist. [Å]** | | ***Spodoptera exigua* cadherin receptor** | |
| --- | --- | --- | --- | --- | --- |
| 1 | A:Leu 337[ N  ] | | 3.23 | | :Asp 674[ OD2] |
| 2 | A:Thr 340[ OG1] | | 3.19 | | :Asp 799[ O  ] |
| 3 | A:Arg 437[ NH1] | | 3.64 | | :Gly 800[ O  ] |
| 4 | A:Asn 484[ OD1] | | 3.53 | | :Ala 543[ N  ] |
| 5 | A:Glu 332[ O  ] | | 3.46 | | :Ala 569[ N  ] |
| 6 | A:Pro 331[ O  ] | | 3.73 | | :Gly 570[ N  ] |
| 7 | A:Asn 343[ OD1] | | 3.28 | | :Arg 738[ NH1] |
| 8 | A:Gly 339[ O  ] | | 3.51 | | :Pro 801[ N  ] |
