# Supplementary material for: In-Silico Determination of Insecticidal Potential of Vip3Aa-Cry1Ac Fusion Protein Against Lepidopteran Targets Using Molecular Docking
Source: Front Plant Sci. 2015 Dec 2;6:1081. doi: 10.3389/fpls.2015.01081 (PMC4667078; doi:10.3389/fpls.2015.01081)
Supplement: Table S5 — Interaction of fusion protein with Spodoptera litura APN receptor. Out of 19 hydrogen bonds present in the docked complex two were less than 2.5 Armstrong in the distance (highlighted). [file Table5.DOCX]

**Table-5:** Interaction of fusion protein with *Spodoptera litura* APN receptor. Out of 19 hydrogen bonds present in the docked complex two were less than 2.5 Armstrong in the distance (highlighted).

| **Sr. No.** | **Fusion protein** | **Dist. [Å]** | | ***Spodoptera litura* APN receptor** | |
| --- | --- | --- | --- | --- | --- |
| 1 | A:Ser 582[ OG ] | | 2.58 | | :Asp  34[ OD1] |
| 2 | A:Ser 582[ OG ] | | 2.65 | | :Tyr  35[ O  ] |
| 3 | A:Ser 290[ OG ] | | 2.49 | | :Phe 877[ O  ] |
| 4 | A:Arg 292[ N  ] | | 3.03 | | :Thr 881[ OG1] |
| 5 | A:Ser 434[ OG ] | | 3.39 | | :Thr 881[ OG1] |
| 6 | A:Ser 293[ N  ] | | 2.49 | | :Thr 885[ OG1] |
| 7 | A:His 295[ ND1] | | 2.69 | | :Thr 885[ O  ] |
| 8 | A:Thr 334[ N  ] | | 3.76 | | :Gly 896[ O  ] |
| 9 | A:Tyr 338[ N  ] | | 3.40 | | :Met 909[ SD ] |
| 10 | A:Thr 340[ OG1] | | 3.62 | | :Met 909[ O  ] |
| 11 | A:Arg 437[ NH1] | | 2.57 | | :Asp 913[ OD1] |
| 12 | A:Arg 437[ NH2] | | 3.37 | | :Asp 913[ OD2] |
| 13 | A:Ser 582[ OG ] | | 3.42 | | :Tyr  35[ N  ] |
| 14 | A:Gln 701[ O  ] | | 3.37 | | :Ser  36[ OG ] |
| 15 | A:Met 341[ O  ] | | 3.40 | | :Gly 543[ N  ] |
| 16 | A:Tyr 306[ OH ] | | 3.52 | | :Glu 544[ N  ] |
| 17 | A:Ser 293[ O  ] | | 2.71 | | :Thr 885[ OG1] |
| 18 | A:Asp 298[ OD2] | | 2.97 | | :Gly 889[ N  ] |
| 19 | A:Glu 332[ O  ] | | 3.05 | | :Ser 897[ OG ] |
