# Supplementary material for: In-Silico Determination of Insecticidal Potential of Vip3Aa-Cry1Ac Fusion Protein Against Lepidopteran Targets Using Molecular Docking
Source: Front Plant Sci. 2015 Dec 2;6:1081. doi: 10.3389/fpls.2015.01081 (PMC4667078; doi:10.3389/fpls.2015.01081)
Supplement: Table S6 — Interaction of fusion protein with Spodoptera litura cadherin receptor. Out of 10 hydrogen bonds present in the docked complex two were less than 3 Armstrong in the distance (highlighted). [file Table6.DOCX]

**Table-6:** Interaction of fusion protein with *Spodoptera litura* cadherin receptor. Out of 10 hydrogen bonds present in the docked complex two were less than 3 Armstrong in the distance (highlighted).

| **Sr. No.** | **Fusion protein** | **Dist. [Å]** | | ***Spodoptera litura* cadherin receptor** | |
| --- | --- | --- | --- | --- | --- |
| 1 | A:Arg 368[ NH1] | | 3.68 | | :Glu 488[ OE1] |
| 2 | A:Tyr 313[ OH ] | | 3.38 | | :Gly 489[ O  ] |
| 3 | A:Tyr 313[ OH ] | | 2.70 | | :Tyr 490[ O  ] |
| 4 | A:Tyr 306[ OH ] | | 3.79 | | :Thr 587[ OG1] |
| 5 | A:Leu 337[ N  ] | | 2.82 | | :Ile 588[ O  ] |
| 6 | A:Ser 441[ OG ] | | 3.59 | | :Arg 743[ O  ] |
| 7 | A:Asn 442[ N  ] | | 3.23 | | :Glu 744[ O  ] |
| 8 | A:Tyr 306[ OH ] | | 2.60 | | :Gln 626[ NE2] |
| 9 | A:Ala 309[ O  ] | | 2.83 | | :Arg 730[ NH2] |
| 10 | A:Glu 332[ O  ] | | 3.56 | | :Thr 608[ OG1] |
